# Using High-Frequency Entropy to Forecast Bitcoin’s Daily Value at Risk

**DOI:** 10.3390/e21020102

**Published:** 2019-01-22

**Authors:** Daniel Traian Pele, Miruna Mazurencu-Marinescu-Pele

**Affiliations:** Department of Statistics and Econometrics, Faculty of Cybernetics, Statistics and Economic Informatics, The Bucharest University of Economic Studies, 010371 Bucharest, Romania

**Keywords:** cryptocurrency, Bitcoin, entropy, value at risk, high-frequency data

## Abstract

In this paper we investigate the ability of several econometrical models to forecast value at risk for a sample of daily time series of cryptocurrency returns. Using high frequency data for Bitcoin, we estimate the entropy of intraday distribution of logreturns through the symbolic time series analysis (STSA), producing low-resolution data from high-resolution data. Our results show that entropy has a strong explanatory power for the quantiles of the distribution of the daily returns. Based on Christoffersen’s tests for Value at Risk (VaR) backtesting, we can conclude that the VaR forecast build upon the entropy of intraday returns is the best, compared to the forecasts provided by the classical GARCH models.

## 1. Introduction

In the past several years, the financial markets witnessed the birth and development of a new assets class, the cryptocurrencies; the starting point was 2008, when the Bitcoin emerged, based on blockchain technology [1]. The cryptocurrencies market is currently one of the most important in the global assets market, with a total market capitalization of approximately 180 billion USD [2]. 

A special focus in the literature is dedicated to the statistical properties and risk behaviour of the cryptocurrencies by comparing them with classical assets like equities or exchange rates. For example, Hu et al. [3] carried out a survey showing that the time series of over 200 cryptocurrencies returns are characterized by large values of kurtosis and volatility and the main risk factor is the Bitcoin itself, which is highly correlated with many altcoins. One implication arising from this paper is that studying the risk behaviour of the Bitcoin is also relevant for the entire cryptocurrency universe.

Zhang et al. [4] highlighted some statistical properties of cryptocurrency returns: heavy tails, volatility clustering and a power-law correlation between price and volume. Chen et al. [5] applied statistical methods (ARIMA, GARCH and EGARCH models) to the CRIX indices family [6], allowing them to observe the volatility clustering phenomenon and the presence of fat tails.

From the risk measures point of view, there is a considerable number of papers dealing with estimation and backtesting of market risk measures on cryptocurrencies. The most popular methods used to estimate VaR or expected shortfall for cryptocurrencies are the ones based on volatility modelling by using GARCH models.

Chu et al. [7] applied GARCH modelling to seven cryptocurrencies (Bitcoin, Dash, Dogecoin, Litecoin, Maidsafecoin, Monero and Ripple) and used the best-fitted model to estimate value at risk (VaR). The main conclusion of their study is that the IGARCH and GJRGARCH models provide the best fit, in terms of modelling the volatility of the most popular and largest cryptocurrencies.

Osterrieder and Lorenz [8] have characterized the risk properties of the Bitcoin exchange rate versus the G10 currencies. By using historical and Gaussian VaR and expected shortfall (ES), they showed that extreme events lead to losses in Bitcoin, which are about eight times higher than what we can expect from the G10 currencies.

All of the GARCH-based models for estimating value at risk are taking into account, in fact, the second moment of the logreturn’s distribution (either daily or intraday). However, the variance, as a measure of statistical uncertainty (as the volatility is a measure of financial risk), captures just a small fraction of the informational content of the distribution of the logreturns.

As Dionisio et al. [9] proved, entropy is a more general measure of uncertainty than the variance or standard deviation, as it may be related to higher-order moments of a distribution, ergo can be more suitable than the variance or volatility to predict value at risk or expected shortfall.

For the classical assets, there are numerous papers showing that entropy has predictive power for the value at risk. For example, Billio et al. [10] prove that entropy has the ability to forecast and predict banking crises, by estimating the entropy of systemic risk measures like marginal expected shortfall and Delta CoVaR.

By using the entropy of the distribution function of intraday returns, Pele et al. [11] proved that entropy is a strong predictor of daily VaR, performing better than the classical GARCH models, for a time series of EUR/JPY exchange rates. Moreover, entropy has a strong explanatory power for the quantiles of the intraday VaR as well as the quantiles of the daily returns.

To the best of our knowledge, very few papers are using entropy in relation to the cryptocurrency market and virtually none of them are using entropy to predict market risk measures for cryptocurrencies.

Apart from the methodology used in [11], instead of using the entropy of the intraday distribution function, we define the entropy of intraday distribution of Bitcoin’s returns, by using symbolic time series analysis (STSA) and producing low-resolution data from high-resolution data.

This approach can be also found in Wilson-Nunn and Zenil [12], who showed that the behaviour of Bitcoin has similarities to stock, gold and silver markets, by using the Shannon entropy [13] on the binary encoded time series of price movements. A similar method is applied by Bariviera et al. [14], who used the Shannon entropy on the Bandt–Pompe time series symbolic encoding of the logreturns on a sample of 12 major cryptocurrencies; their results show that the majority of the cryptocurrencies exhibit a similar behaviour, compatible with a persistent stochastic process with fractal dimension between 1.3 and 1.5. 

By using the same Bandt–Pompe time series symbolic encoding, Sensoy [15] studied the weak-form efficiency of Bitcoin prices at a high-frequency level by using permutation entropy, finding that the volatility has a significant negative effect on the informational efficiency of Bitcoin prices.

However, the Bandt-Pompe time series symbolic encoding [16] has some methodological weaknesses. As shown in Zunino et al. [17], there are some special situations when this symbolic encoding leads to false conclusions regarding the underlying structures of the analysed time series. 

In this paper, we are using the entropy of intraday Bitcoins returns, through symbolic time series analysis (STSA), in order to forecast daily value at risk (VaR), starting from the established fact [18] that entropy, as a measure of complexity, is associated with periods of low returns and high volatility, for the classical stock market.

Bitcoin appears to be the perfect candidate for testing this hypothesis on the cryptocurrencies market, as shown in Stavroyiannis [19]. By using the GARCH modelling followed by a filtered historical simulation in order to estimate daily value at risk and expected shortfall for Bitcoin, Ethereum, Litecoin, and Ripple and by comparing the estimation results with the ones obtained for the S&P500 Index, the author concludes that the digital currencies are subject to higher risk: “Bitcoin is a highly volatile currency violating the value-at-risk measures more than the other assets”.

Our results show that entropy is a predictor of the crisis periods in the evolution of the Bitcoin exchange rates, in line with the findings from Soloviev and Belinskij [20], who used permutation entropy as a measure of complexity. 

The main objective of this paper is to study the link between the entropy of the high-frequency intraday Bitcoin’s returns and daily VaR to demonstrate its VaR-forecasting ability. We are comparing the forecast abilities of several models, including historical simulation and GARCH models and we evaluate the statistical accuracy of one-day-ahead VaR estimates by means of the unconditional coverage test, the independence test and the conditional coverage test (Christoffersen, [21]). 

In light of the findings from the literature, our contribution to the studies dealing with the market risk associated to cryptocurrencies is mostly empirical. By using the entropy of intraday Bitcoin’s returns as a predictor for the Bitcoin’s daily value at risk, we prove that entropy has a better forecasting ability than the classical GARCH models for Bitcoin’s daily VaR. 

Our results add to the findings from a recent paper (Colucci [22]), where several models that forecast ex-ante Bitcoin one-day value at risk (VaR) are compared: parametric normal, historical simulation, historical filtered bootstrap, extreme value theory historical filtered bootstrap, Gaussian and Student-t GARCH models. The performance of all VaR models is validated using both statistical accuracy and efficiency evaluation tests. One conclusion of the study is to avoid the use of the parametric normal and the standard historical simulation approach, due to their limitations in value at risk estimation. Another important conclusion of their study is that both normal and Student-t GARCH models are useful for estimating Bitcoin value at risk: the normal GARCH from the investors’ point of view and the Student-t GARCH from the regulators’ point of view.

Our paper extends these results, by proving that the entropy has better forecasting ability for the ex-ante Bitcoin one-day value at risk (VaR) than the classical GARCH models.

The rest of the paper is organized as follows: Section 2 details the methodology; Section 3 presents the dataset and the empirical results and Section 4 concludes.

## 2. Methodology

The methodology used in this paper has two layers: first, we define the Shannon entropy [13] of the intraday Bitcoin’s returns, by using symbolic time series analysis (STSA) [23] and show how the probability of extreme losses is related to the entropy.

Second, we investigate the VaR-forecasting ability of the entropy of the intraday Bitcoin’s returns, by comparing the forecasting ability of several models and by using the appropriate backtesting tests.

### 2.1. Entropy and Bitcoin Daily Prices

Entropy is both a measure of uncertainty and complexity of a system, with numerous applications in physics (the second principle of thermodynamics), in information theory, in biology (DNA sequence complexity), medicine and economics (complexity of a system).

If *X* is a discrete random variable, with probability distribution $X:\left(\begin{array}{l} x_{1}\ldots x_{n}\\ P_{i}\ldots P_{n} \end{array}\right)$, where $p_{i}=P(X=x_{i})$, $0\leq p_{i}\leq 1$ and $\sum_{i}p_{i}=1$, then the Shannon Information Entropy is defined as follows:

(1) $$S(X)=-\sum_{i}p_{i}\log_{2}p_{i}.$$

For the uniform distribution, the Shannon entropy reaches its maximum: $S(X)=-\sum_{i}\left(1/n)\log_{2}(1/n\right)=\log_{2}n$, while the minimum value is attained for a distribution like the following: $X:\left(\begin{array}{ccc} x_{1} & \ldots & x_{n}\\ 1 & \ldots & 0 \end{array}\right)$, for which S(X)=0.

In other words, higher levels of entropy are obtained for situations with higher uncertainty and lower levels of entropy correspond to situations with lower uncertainty.

In order to define the entropy of intraday distribution of Bitcoin’s returns, we are using symbolic time series analysis (STSA), by producing low-resolution data from high-resolution data [23].

Let $P_{t,v}$ be the intraday price of the asset (in our case, the Bitcoin) recorded in day *t*, with t∈[1,T] at trading time ν, with $\nu \in [1,N_{t}]$, *N_t_* being the number of trading moments, equally-spaced, from day *t*.

Then the intraday logreturn can be defined as:

(2) $$r_{t,\nu }=\log P_{t,v}-\log P_{t,v-1}.$$

The symbolic representation of the time series of intraday logreturns can be done through the transformation $r_{t,\nu }\overset{STSA}{\to }s_{t,\nu }$, where:

(3) $$s_{t,\nu }=\left\{ \begin{array}{l} 1,\;if\;r_{t,\nu }<0\\ 0,\;if\;r_{t,\nu }\geq 0 \end{array}\right..$$

Through this transformation, the time series of intraday logreturns is symbolically represented by a binary sequence of 0 and 1, showing the moments of prices going upward or downward.

Using the intraday prices for one trading day t, one can compute the probability $p_{t}=\mathrm{Pr}(s_{t,\nu }=1)$. 

Then the Shannon entropy for the trading day t is:

(4) $$S_{t}=-p_{t}\log_{2}p_{t}-(1-p_{t})\log_{2}(1-p_{t}).$$

Our working hypothesis is that the daily exchange rate of Bitcoin is correlated to the daily entropy of intraday returns through the following relationship:

(5) $$E(P_{t})=\exp[\beta_{0}+\beta_{1}S_{t}].$$

The reason behind the Equation (5) is that the fluctuations in the intraday market, captured by entropy, should also be visible in the fluctuation of daily exchange rates. As it could be seen in Section 3, this working hypothesis was confirmed by the empirical data.

### 2.2. Entropy as a Predictor of Extreme Values of Daily Returns Distribution

Entropy can be seen as a measure of market complexity; as shown in [18], when the price exhibits a predominant trend (upwards or downwards), the level of certainty is high, and such periods are described by lower values of entropy.

Our working hypothesis is that the likelihood of extreme negative daily returns can be explained by lower values of entropy. To verify this hypothesis, we estimate the following logistic regression model:

(6) $$P(Y_{t}^{*}=1)=\frac{\exp(b_{0}+b_{1}S_{t})}{1+\exp(b_{0}+b_{1}S_{t})}.$$

In the above equation, we have:

- $Y_{t}^{*}$ is the indicator of the lower tails of the daily logreturn’s distribution: $Y_{t}^{*}=\left\{ \begin{array}{l} 1,\;if\;r_{t}<-VaR_{\alpha }\\ 0,\;if\;r_{t}\geq -VaR_{\alpha } \end{array}\right.$, where VaR at significance level α is defined by the following equation:
  (7) $$\mathrm{Pr}(r_{t}<-VaR_{\alpha })=\alpha .$$
- $S_{t}$ is the Shannon information entropy at day *t,* quantified by Equation (4).

A performance indicator of the logistic regression model is defined by comparing the likelihood function of estimated model with the likelihood function of the model when the exogenous variable is removed.

One can define *pseudo-*$R^{2}$ as a measure of model’s performance [24]:

(8) $$R^{2}=1-\exp\{2[\log L(M)-\log L(0)]/n\}.$$

where L(M) and L(0) are the likelihood functions of the model, with and without the exogenous variable. 

Rewriting the expression (7) as $-\log(1-R^{2})=2[\log L(M)-\log L(0)]/n$, this could be interpreted as the surplus of information due to explanatory variable. As $R^{2}$ will never reach 1, not even for a perfect model, the following adjustment has been made:

(9) $$R_{adj}^{2}=R^{2}/[1-\exp(2\log L(0)/n)].$$

### 2.3. Forecasting Daily VaR Using Entropy

In order to forecast the daily VaR using entropy we are following the approach used in [11].

Firstly, we estimate the following quantile regression model, by using the Shannon entropy of the distribution of intraday returns as explanatory variable:

(10) $$Q_{r,t}(\tau )=b_{0}+b_{1}S_{t}+\varepsilon_{t}.$$

where the quantile of the returns is denoted by $Q_{r,t}(\tau )=\underset{s\leq t}{\inf}\left\{r_{s},F(r_{s})\geq \tau \right\}$.

The aim of this quantile regression model is to explain the relationship between the quantiles of the distribution of the daily logreturns and the Shannon entropy of the distribution of the intraday returns.

Secondly, we use a rolling window approach and the quantile regression model below to forecast the daily VaR based on the lagged entropy of intraday returns:

(11) $$Q_{r,t}(\alpha )=b_{0}+b_{1}S_{t-1}+\eta_{t}.$$

Estimating the Equation (11) on the time interval [*k*+1, *k*+*w*], the estimates $b_{0}^{k}$ and $b_{1}^{k}$ are obtained. 

Then the forecast of VaR for the next trading day is given by the following equation:

(12) $${\hat{VaR}}_{\alpha ;k+w+1}=-b_{0}^{k}-b_{1}^{k}S_{k+w}.$$

where:

- *w* is the length of the rolling window;
- t∈{k+1,…,k+w}, k∈{0,…,T−w+1};
- *T* is the number of daily returns.

One can extend the Equation (11) to include an autoregressive term as below:

(13) $$Q_{r,t}(\alpha )=b_{0}+b_{1}S_{t-1}+b_{2}Q_{r,t-1}(\alpha )+\nu_{t}.$$

By estimating the Equation (13) using a rolling window approach, then the next day forecast of VaR can be computed as:

(14) $${\hat{VaR}}_{\alpha ;k+w+1}=-b_{0}^{k}-b_{1}^{k}S_{k+w}+b_{2}^{k}{\hat{VaR}}_{\alpha ;k+w}.$$

In order to assess the performance of the entropy-based VaR, we test the forecasting ability of the following models, estimated using the rolling window *w*:

- Historical VaR forecasts;
- Normal GARCH(1,1) VaR forecasts;
- Student’s t-GARCH(1,1) VaR forecasts;
- Entropy-based VaR forecasts, given by forecasting Equation (12);
- Entropy-based autoregressive VaR forecasts, given by forecasting Equation (14).

The comparison between these five models is done based on the Christoffersen’s tests [21], detailed below.

- The LR Test of Unconditional Coverage:
  Let ${\hat{VaR}}_{t}$ be the forecasted VaR and let $r_{t}$ be the daily logreturn. If we define $I_{t}=\left\{ \begin{array}{l} 1,\;if\;r_{t}<-{\hat{VaR}}_{t}\\ 0,\;otherwise \end{array}\right.$, then the hypotheses being tested are: $\left\{ \begin{array}{l} H_{0}:E(I_{t})=\alpha \\ H_{A}:E(I_{t})\neq \alpha \end{array}\right.$.
  The test statistic is defined as:
  $$LR_{uc}=-2\log\frac{L(\alpha )}{L(\hat{\alpha })}=-2\log\frac{\alpha^{n_{0}}{\left(1-\alpha \right)}^{n-n_{0}}}{{\hat{\alpha }}^{n_{0}}{\left(1-\hat{\alpha }\right)}^{n-n_{0}}}\approx \chi^{2}(1),\mathrm{where}$$
  $$\hat{\alpha }=\frac{n_{0}}{n}=\mathrm{Pr}(I_{t}=0).$$
- The LR Test of Independence:
  Let $I_{t}$ be a first-order Markov chain with transition probability matrix:
  $$\Pi_{1}=[\begin{array}{l} 1-\pi_{01}\;\pi_{01}\\ 1-\pi_{11}\;\pi_{11} \end{array}],\;\mathrm{where}\;\pi_{ij}=\mathrm{Pr}(I_{t}=i|I_{t-1}=j).$$
  The test statistic is defined as $LR_{i}=-2\log\frac{L({\hat{\Pi }}_{2})}{L({\hat{\Pi }}_{1})}\approx \chi^{2}(1),\mathrm{where}$
  $L(\Pi_{1})={\left(1-\pi_{01}\right)}^{n_{00}}{\pi_{01}}^{n_{01}}{\left(1-\pi_{11}\right)}^{n_{10}}{\pi_{11}}^{n_{11}}$, $L(\Pi_{2})={\left(1-\pi_{2}\right)}^{n_{00}+n_{10}}{\pi_{2}}^{n_{01}+n_{11}}$, with
  $\pi_{2}=\frac{n_{01}+n_{11}}{n_{01}+n_{00}+n_{10}+n_{11}}$.
- The Joint Test of Coverage and Independence
  The test statistic is given by $LR_{full}=-2\log\frac{L(\alpha )}{L({\hat{\Pi }}_{1})}\approx \chi^{2}(2)$.

## 3. Data and Empirical Results

In order to illustrate the use of the entropy of the distribution of intraday returns in forecasting the daily VaR of Bitcoin, we consider the BTC/USD exchange rate, sourced from https://www.cryptodatadownload.com, for the time period 10/08/2015–10/02/2018. The database used for the estimation has two components: (1) intraday prices (1090 transaction days and 1,569,600 minute-by-minute intraday observations); and (2) daily prices (1090 daily observations). 

The dataset covers three years of daily transaction data and the starting point was chosen so that the time period is relevant in terms of transaction volume and liquidity. As documented in [25], before 2015, “the total market capitalization of the cryptocurrency market had been less than 16 billion dollars, the daily transaction volume was also low and the liquidity was not good”. 

**Figure 1:**
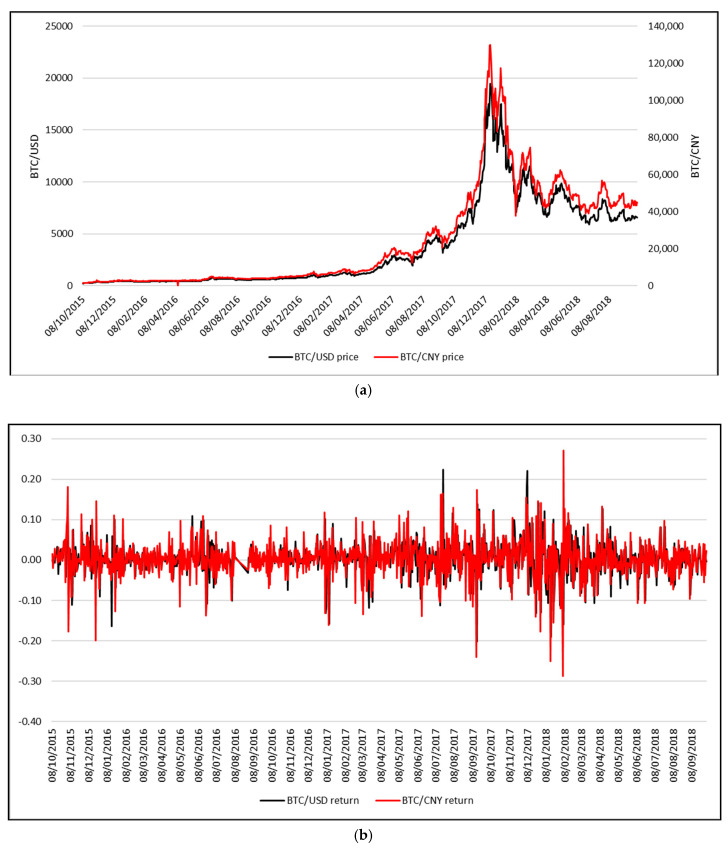
(**a**) Bitcoin average daily price (USD) vs. (CNY); (**b**) Bitcoin daily logreturn (USD) vs. (CNY).

Most of the published papers in this field are using the BTC/USD exchange rates, although it may be worth exploring the time series of BTC/CNY exchange rates, as 74 percent of the computing power needed to mine and verify Bitcoin transactions currently resides in China, according to a recent study by Kaiser et al. [26]. However, as shown in Figure 1a,b, the time series of BTC/USD and BTC/CNY follow the same pattern, both in terms of absolute prices and daily logreturns.

### 3.1. Entropy and Bitcoin Daily Price

**Figure 2:**
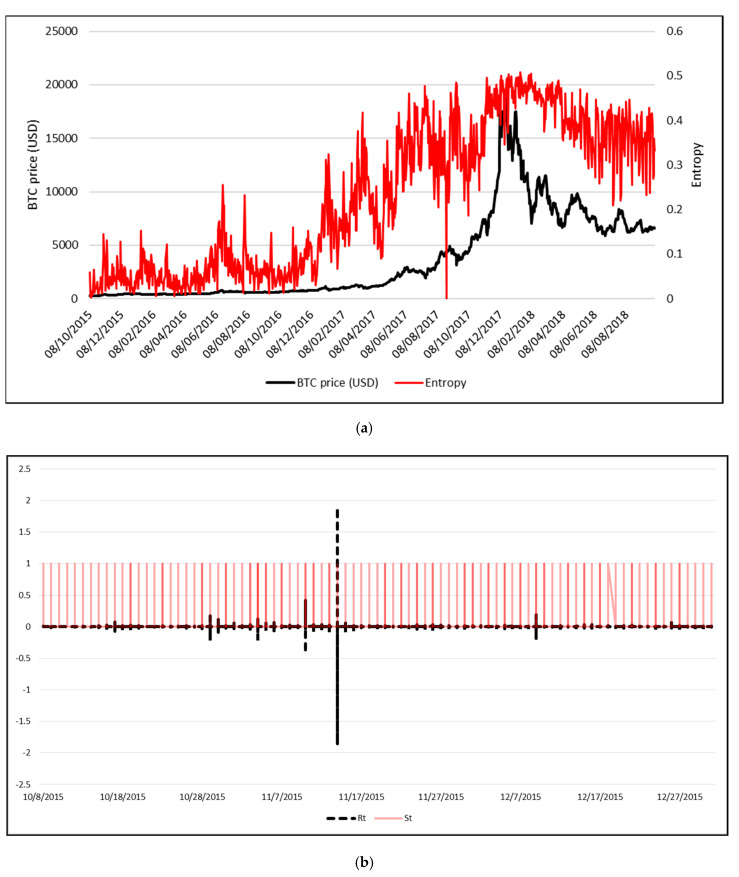

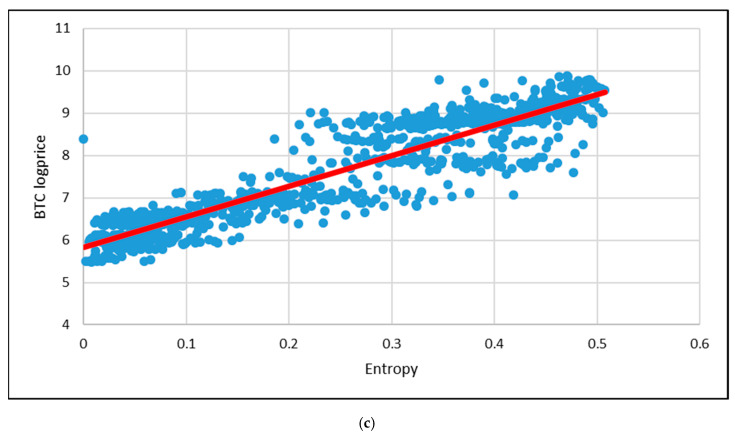
(**a**) Bitcoin average daily price (USD) vs. entropy; (**b**) Symbolic representation of the time series of intraday minute-by-minute logreturns for 2015; (**c**) Bitcoin daily logprice vs. entropy.

Figure 2a presents a comparison of the daily entropy and average daily exchange rate of Bitcoin, while Figure 2b presents the correlation between the daily logprice of the Bitcoin and entropy. The entropy was computed using the symbolic time series analysis (STSA) applied to the intraday minute-by-minute Bitcoin’s logreturns.

As shown in Figure 2a, the Bitcoin’s high price regime encountered at the end of 2017 was associated to high values of the entropy of intraday returns. More precisely, on December 17, 2017, when the Bitcoin price reached its historical maximum (approx. 19,476 USD), the estimated entropy was 0.47. In relative terms, the Bitcoin price increased by 10% compared to the previous trading day (from 17760 USD to 19476 USD), while the entropy increased by 36% (from 0.35 to 0.47).

It appears to be a very strong positive correlation between the daily logarithmic price of Bitcoin and the entropy of intraday returns, as confirmed by the following regression model:

(15) $$\log P_{t}=\beta_{0}+\beta_{1}S_{t}+\varepsilon_{t}.$$

The model (15) is another representation of the Equation (5): $E(P_{t})=\exp[\beta_{0}+\beta_{1}S_{t}]$, relating the Bitcoin’s logprice to the entropy.

The estimation results for the model (15) are shown in Table 1.

The $R_{adj}^{2}$ estimate indicates that the entropy is strongly linked to the daily exchange rate, showing that it has forecasting power over the price dynamics of Bitcoin. Moreover, the coefficient of the entropy is positive and significant, pointing out that the Bitcoin’s price dynamics might be driven by market uncertainty.

However, this model may not be so accurate, due to the well-known weaknesses of the OLS applied to times series. In the following subsection, the conclusions obtained from the OLS model are strengthened by using alternative modelling techniques, like logistic regression and quantile regression. 

### 3.2. Entropy as a Predictor of Extreme Values of Daily Returns Distribution—Results

We estimated the model from the Equation (6) by using $VaR_{\alpha }$ as a threshold for both α = 0.01 and α = 0.05 (see Table 2).

Analysing the results of the estimation, we can see that the entropy is positively correlated to the likelihood of extreme values of daily returns. More precisely, if the entropy of intraday returns increases, then we have a dramatic increase of the probability of an extreme negative Bitcoin return.

The low values of the adjusted R-squared for the estimated logistic regression model may raise some questions regarding the quality of the model. However, the purpose of this analysis is not to find the most comprehensive model in terms of explanatory power, but to prove that the entropy has a significant impact on the likelihood of extreme negative Bitcoin’s returns. In this context, low values of the adjusted R-squared may indicate a real problem only if we want to use this model to obtain accurate point predictions; yet, this is out of our scope of work and the model is still valid, despite its low R-squared values. We will interpret this result as an argument for the fact that entropy, as a measure of market uncertainty, can give an indication about the presence of an extreme negative event in the distribution of Bitcoin’s daily logreturns.

This result relates to some known facts from the literature; for example, Risso [18] and Pele [27] who have found that entropy is a predictor of stock market crisis, while Soloviev and Belinskij [20] proved the same thing for the cryptocurrencies market by using a different entropy measure.

Further, we estimated the quantile regression model (10) in order to quantify the effect of the entropy on the quantiles of daily logreturns distribution.

The Equation (10) was estimated for τ = 0.01, 0.05 and 0.10; the estimation results are presented in Table 3. 

**Figure 3:**
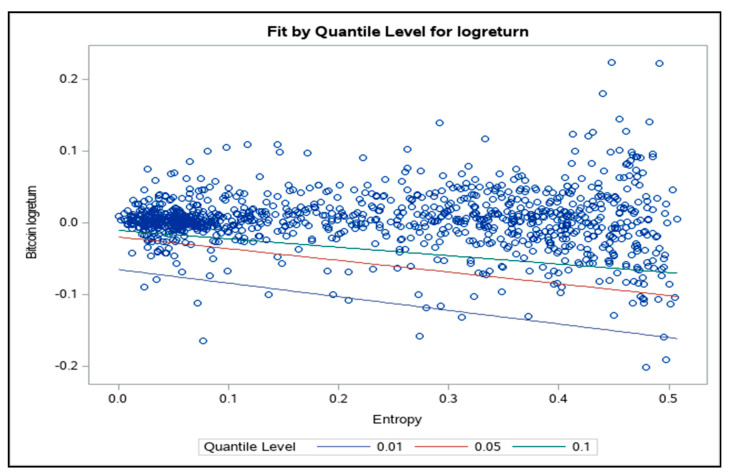
Quantile regression results.

The relationship between the quantiles and the entropy of intraday returns for Bitcoin is negative and significant. Lower values of the entropy of the distribution of intraday returns generally correspond to higher quantiles for the distribution of daily returns (see Figure 3).

In other words, high values of entropy (high uncertainty) are associated with low values of daily logreturns, i.e., high values of VaR. This result is consistent with the one documented on the foreign exchange market [11], where the relationship between the quantile of the logreturns distribution and the entropy was found to be negative and significant.

### 3.3. Forecasting Daily VaR Using Entropy

**Figure 4:**
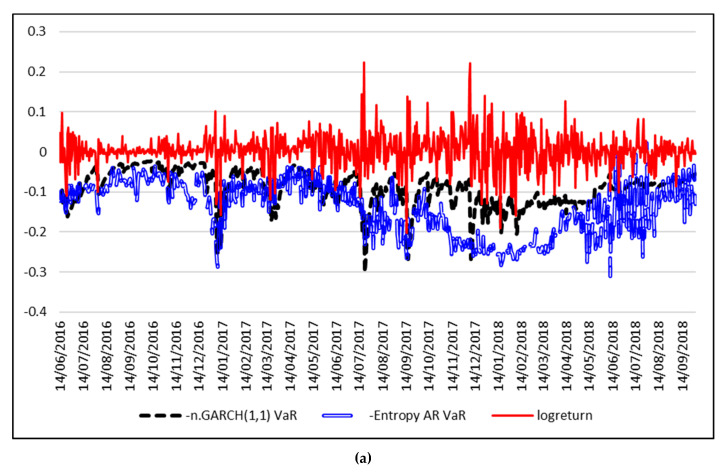

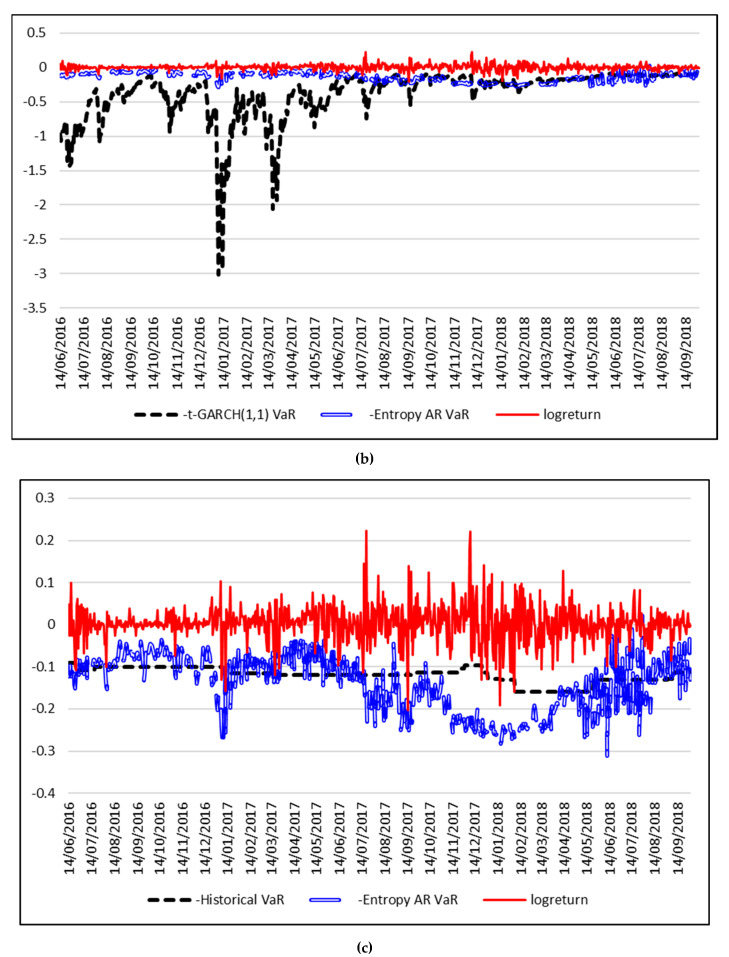
Forecasts for daily 1% VaR using entropy. (**a**) Entropy AR-VaR vs. n.GARCH(1,1) VaR. (**b**) Entropy AR-VaR vs. t.GARCH(1,1) VaR. (**c**) Entropy AR-VaR vs. Historical VaR.

**Figure 5:**
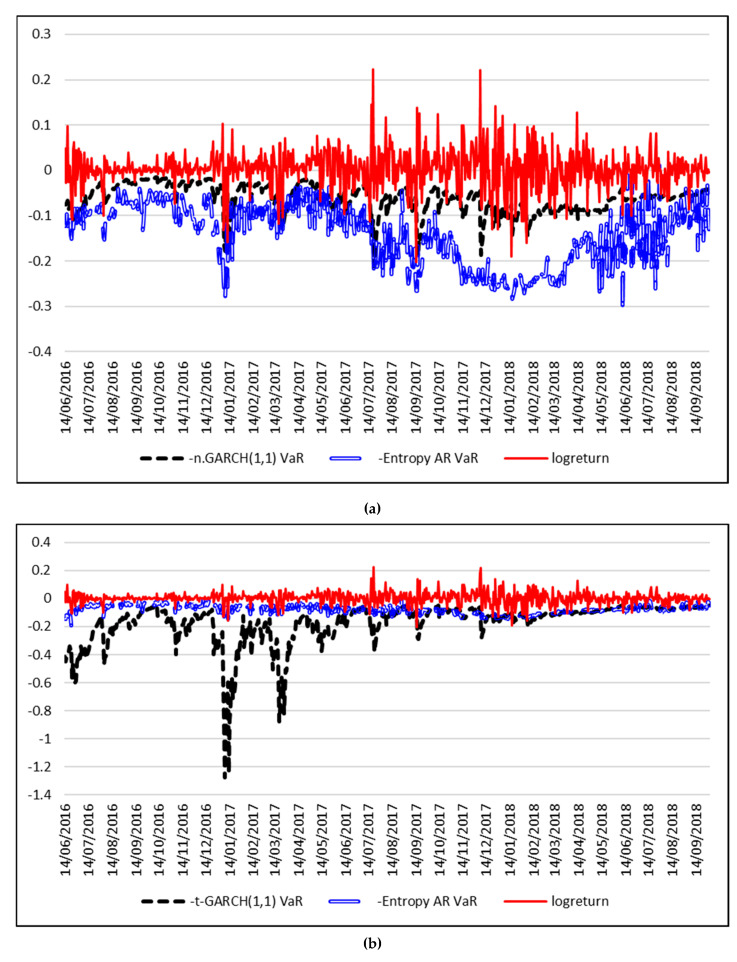

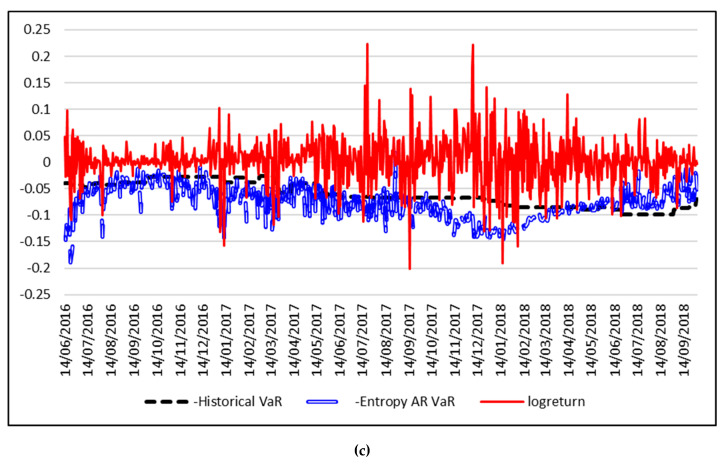
Forecasts for daily 5% VaR using entropy. (**a**) Entropy AR-VaR vs. n.GARCH(1,1) VaR. (**b**) Entropy AR-VaR vs. t.GARCH(1,1) VaR. (**c**) Entropy AR-VaR vs. Historical VaR.

In order to forecast VaR, the Equations (12) and (14) are estimated using a rolling window approach, with a window of length *w* = 250 trading days. The time series of daily logreturns and the forecasted VaR for 1% are depicted in Figure 4, while Figure 5 shows the results for VaR 5%. 

At 1% significance level, the VaR forecasts based on the Entropy-AR model outperforms the Historical VaR and the Gaussian-based VaR and even the VaR based on the GARCH model with Student residuals. The forecasted 1% VaR based on the Student GARCH model is extremely unrealistic, compared to the actual time series of Bitcoin logreturns, with estimated values of 300%. The same situation is encountered at a 5% significance level, where the best forecast is also given by the model based on the entropy of intraday Bitcoin returns.

The backtesting results of the VaR forecasts for the models given in Section 2.2, for α = 1% and α = 5% are presented in Table 4 and Table 5. 

Regarding the 1% VaR forecasts, it can be seen—looking at the second column, which gives the probability that the returns are below the negative of the VaR—that the entropy-based AR VaR forecast model (with a probability of 0.0024) provides the highest VaR forecasts on average. Looking at the results of the Christoffersen’s tests (unconditional, independence and full test), it can be seen that the entropy-based AR VaR forecast model is the only one that passes all the three tests with a 99% confidence level.

Regarding the 5% VaR forecasts, the entropy-based AR VaR forecast model passes only the unconditional and full Christoffersen’s tests (like all the other models), yet it has the lowest probability that the returns are below the negative of the VaR. 

One concern may arise from the fact the LR statistics are identical for the VaR estimates using the Student GARCH(1,1) model and the entropy model. This occurs because of the nature of Christoffersen’s tests, as they take into account only the exceedances of the -VaR over the real daily logreturn, not the actual value of the error.

For the particular situation of the year 2018, when the entire cryptocurrencies market had dramatically declined, we report in Table 6 the results of the 5% VaR forecast backtest.

**Figure 6:**
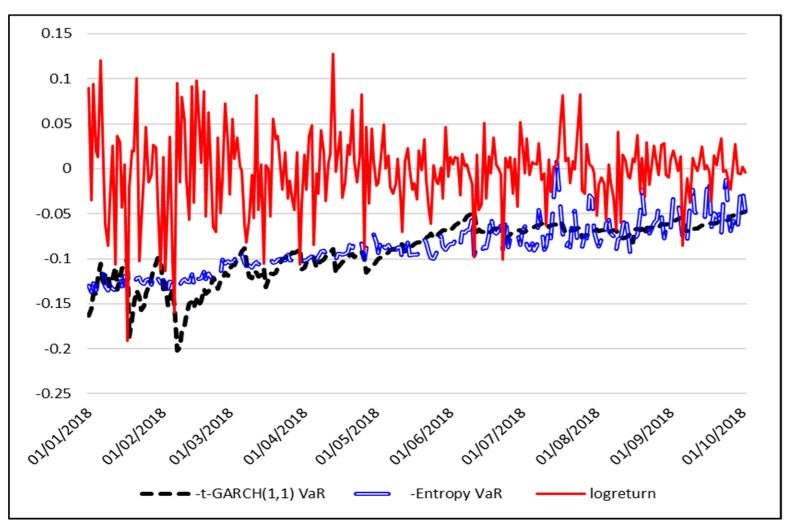
Forecasts for 2018 daily 5% VaR using entropy.

For 2018, the 5% VaR forecast based on the entropy VaR model has similar performance to the t-GARCH (1,1) VaR model in terms of the number of violations, as could also be seen in Figure 6. However, the entropy VaR model superior power is proved by the Christoffersen test, at 90% confidence level.

## 4. Conclusions

This paper investigates the relationship between entropy and Value-at-Risk for the Bitcoin, the most important representative of a new class of assets, the cryptocurrencies. Using high frequency data for the Bitcoin, we estimated the entropy of intraday distribution of logreturns, through symbolic time series analysis (STSA), producing low-resolution data from high-resolution data. The conclusions of our analysis highlight the idea that entropy has a good explanatory power for the price and return behaviour of Bitcoin. 

First, we confirmed the hypothesis that the daily exchange rate of Bitcoin is correlated to the daily entropy of intraday returns. There is a very strong positive correlation between the daily logarithmic price of Bitcoin and the entropy of intraday returns, showing that entropy has forecasting power over the price dynamics of Bitcoin. Moreover, the coefficient of the entropy is positive and significant, pointing out that Bitcoin’s price dynamics may be driven by market uncertainty. This finding enriches the literature dealing with the analysis of cryptocurrencies, pointing out that the entropy has a significant negative effect on the informational efficiency of Bitcoin prices (in [15] the volatility was found to have a similar effect).

Second, the hypothesis that the likelihood of extreme negative daily returns of Bitcoin can be explained by lower values of entropy was confirmed through empirical analysis. By analysing the results of the estimation, we can conclude that the entropy is positively correlated to the likelihood of extreme values of daily returns. Our results show that entropy is a predictor of the crisis periods in the evolution of the Bitcoin exchange rates, in line with the findings from Soloviev and Belinskij [20].

Third, our findings show that entropy has a strong explanatory power for the quantiles of the distribution of the Bitcoin daily returns. Based on Christoffersen’s tests for VaR backtesting, we can conclude that the VaR forecast builds upon the entropy of intraday returns and is the best among the forecasts provided by the classical GARCH models.

At 1% significance level, the VaR forecasts based on entropy-AR model outperforms the historical VaR, the Gaussian-based VaR and the VaR based on the GARCH model with Student residuals. The same situation is encountered at 5% significance level, where the best forecast is also given by the model based on the entropy of intraday Bitcoin returns.

Our results extend the findings from the literature (Colucci [22]) by proving that entropy has a better forecasting ability for the ex-ante Bitcoin one-day value at risk (VaR) than the classical GARCH models.

## Figures and Tables

**Table 1:** Estimation results of the model (15).

Variable	Estimate
Intercept	5.839 ***
	(0.017)
Entropy	7.212 ***
	(0.076)
$R_{adj}^{2}$	0.868

Note: *** denotes statistical significance at 99% confidence level; standard errors in ().

**Table 2:** Estimation results of logistic regression.

Parameter	*α = 0.01*	*α = 0.05*
*b*_0_	−6.253 *** (0.901)	−4.495 *** (0.384)
*b*_1_	5.340 *** (2.395)	5.328 *** (1.038)
Odds Ratio	208.513	206.026
$\mathrm{Pseudo}-R_{adj}^{2}$	0.057	0.090

Note: *** denotes statistical significance at 99% confidence level; standard errors in ().

**Table 3:** Quantile regression results.

	τ = 1%	τ = 5%	τ = 10%
*S_t_*	−0.190 ***	−0.163 ***	−0.117 ***
	(0.068)	(0.022)	(0.017)

Note: *** denotes statistical significance at 99% confidence level; standard errors in ().

**Table 4:** 1% VaR forecast backtest results.

Model	$\mathrm{Pr}(R_{t}<-{\hat{VaR}}_{t})$	$LR_{uc}\;\mathrm{Test}$	$LR_{i}\;\mathrm{Test}$	$LR_{full}\;\mathrm{Test}$
Historical VaR	0.014	1.375	6.766 ***	8.142 **
n.GARCH(1,1) VaR	0.025	13.476 ***	0.272	13.748 ***
t-GARCH(1,1) VaR	0.003	4.657 **	6.489 **	11.147 ***
Entropy VaR	0.003	4.657 **	6.489 **	11.147 ***
Entropy AR VaR	0.002	7.108 ***	7.837 ***	14.945 ***

Note: ** and *** denotes statistical significance at 90%, 95% and 99% confidence level, respectively.

**Table 5:** 5% VaR forecast backtest results.

Model	$\mathrm{Pr}(R_{t}<-{\hat{VaR}}_{t})$	$LR_{uc}\;\mathrm{Test}$	$LR_{i}\;\mathrm{Test}$	$LR_{full}\;\mathrm{Test}$
Historical VaR	0.061	2.337	3.924 **	6.262 **
n.GARCH(1,1) VaR	0.060	1.905	4.276 **	6.182 **
t-GARCH(1,1) VaR	0.017	24.014 ***	1.049	25.063 ***
Entropy VaR	0.020	20.023 ***	0.720	20.744 ***
Entropy AR VaR	0.019	21.955 ***	0.874	22.830 **

Note: ** and *** denotes statistical significance at 90%, 95% and 99% confidence level, respectively.

**Table 6:** 5% VaR forecast backtest results for 2018.

Model	$\mathrm{Pr}(R_{t}<-{\hat{VaR}}_{t})$	$LR_{uc}\;\mathrm{Test}$	$LR_{i}\;\mathrm{Test}$	$LR_{full}\;\mathrm{Test}$
Historical VaR	0.047	0.038	0.222	0.260
n.GARCH(1,1) VaR	0.058	0.387	0.005	0.391
t-GARCH(1,1) VaR	0.029	2.917 *	1.148	4.064
Entropy VaR	0.026	4.170 **	1.534	5.705 *
Entropy AR VaR	0.033	1.921	0.833	2.754

Note: * and ** denotes statistical significance at 90%, 95% and 99% confidence level, respectively.
